# Exacerbated impairments in neuromuscular function when two bouts of team sport match simulations are separated by 48 h

**DOI:** 10.1113/EP091419

**Published:** 2023-10-09

**Authors:** Joseph Hubbard, Jade Pitcairn, Callum G. Brownstein

**Affiliations:** ^1^ School of Biomedical, Nutritional, and Sport Sciences Faculty of Medical Sciences Newcastle University Newcastle upon Tyne UK

**Keywords:** contractile function, fatigability, muscle damage, soccer, team sports, voluntary activation

## Abstract

Intermittent team sports, involving high metabolic and mechanical demands, elicit prolonged impairments in neuromuscular function which persist for ∼48–72 h. Whether impairments in neuromuscular function are exacerbated when such exercise is repeated with incomplete recovery is unknown. This study assessed the neuromuscular, heart rate and metabolic responses to two bouts of ∼90 min modified team sport match simulations separated by 48 h in 12 competitive football players. Before and 2 min after both bouts, knee extensor isometric maximal voluntary contraction (MVC), contractile function (*Q*_tw,pot_) and voluntary activation (VA) were measured. Heart rate (HR), sprint time, blood lactate and glucose were measured throughout both bouts. MVC was reduced relative to baseline at post‐bout 1 (21 ± 12%; *P* = 0.003) and pre‐bout 2 (14 ± 11%; *P* = 0.009), and was lower post‐bout 2 (33 ± 14%; *P* < 0.001) relative to post‐bout 1 (*P =* 0.036). *Q*_tw,pot_ was reduced post‐bout 1 (30 ± 11%; *P* < 0.001) and pre‐bout 2 (9 ± 6%; *P* = 0.004), and was not different post‐bout 2 (28 ± 8%; *P* < 0.001) relative to post‐bout 1 (*P* = 0.872). VA was reduced post‐bout 1 (8 ± 7%; *P* = 0.023), recovered pre‐bout 2 (*P* = 0.133) and was lower post‐bout 2 (16 ± 7%; *P* < 0.001) relative to post‐bout 1 (*P* = 0.029). Total sprint time was longer, and HR, blood lactate and glucose were lower during bout 2 than bout 1 (*P* ≤ 0.021). Thus, impairments in neuromuscular function are exacerbated when high‐intensity intermittent exercise is performed with incomplete recovery concurrent with accentuated reductions in VA. The lower blood lactate and glucose during the second bout might be due, at least in part, to reduced glycogen availability upon commencing exercise and consequently a greater reliance on glucose extraction.

## Abstract (highlights)

- **What is the central question of this study?**
  There is limited evidence on whether impairments in neuromuscular function are exacerbated when prolonged high‐intensity intermittent exercise is repeated with incomplete recovery: what are the neuromuscular consequences of performing two bouts of a modified team sport match simulations separated by 48 h?
- **What is the main finding and its importance?**
  Impairments in knee extensor force generating capacity are exacerbated concurrent with accentuated reductions in nervous system activation of muscle when prolonged high‐intensity intermittent exercise is repeated with 48 h recovery.

## 1 INTRODUCTION

In recent decades, a plethora of research has assessed the neuromuscular consequences of dynamic whole‐body locomotor exercise (e.g., running and cycling) of varying durations, intensities and modalities (for review see Brownstein et al., 2021). The prevailing approach to the study of neuromuscular fatigability in response to locomotor exercise is to perform assessments of neuromuscular function before and immediately following a single bout of exercise (Place & Millet, 2020). However, the training and competition schedule among athletes is such that they regularly perform exercise on multiple days per week having recovered incompletely following a previous exercise bout. The neuromuscular consequences of performing exercise with incomplete restoration of neuromuscular function following a bout of exercise in the preceding days is unknown. For example, it is unclear whether and to what extent impairments in neuromuscular function are exacerbated when exercise is performed with incomplete recovery, and if so, through which mechanisms.

A suitable model to assess whether impairments in neuromuscular function are exacerbated when exercise is performed with incomplete recovery is to employ prolonged, metabolically and mechanically strenuous exercise that results in persistent reductions in neuromuscular function, and to repeat a similar bout of exercise prior to complete restoration of neuromuscular function. One modality of exercise which fits these characteristics is high‐intensity intermittent, team sport‐related activity. Indeed, team sports such as association football involve substantial metabolic demands owing to the prolonged distances covered (9–12 km in elite level players), and the volume of high‐intensity running (2–3 km covered at high running speeds >18 km/h) and sprinting (∼500 m) (Barnes et al., 2014; Mohr et al., 2003). Consequently, high‐intensity intermittent team sports results in metabolic perturbations which can take days to resolve. For example, muscle glycogen stores are reduced by ∼50% post‐match and remain below baseline for up to 72 h post‐match (Gunnarsson et al., 2013; Krustrup et al., 2011). In addition to these metabolic demands, high mechanical stress is placed on the musculoskeletal system through the frequent and intense decelerations and diverse range of movements such as jumping, landing, tackling and changing direction (Dalen et al., 2016; de Hoyo et al., 2016; Harper et al., 2019). The large eccentric component associated with many of these activities elicits substantial muscle damage which persists for a number of days post‐exercise. For example, elevated markers of muscle damage and inflammation have been reported beyond 72 h following football match‐play (Ascensão et al., 2008; Ispirlidis et al., 2008; Magalhães et al., 2010). Consequently, the high metabolic and mechanical demands associated with team sport activity lead to metabolic and histological perturbations which can take several days to recover.

The persistent metabolic and histological perturbations in the days following team sport activity likely contribute to the prolonged impairments in neuromuscular function, and perceptions of fatigue and soreness following match‐play. For example, glycogen depletion (Ørtenblad et al., 2013) and muscle damage (Allen, 2001; Goodall, Thomas, Barwood, et al., 2017) are associated with impairments in neuromuscular function which manifest through reductions in the maximal force generating capacity of the involved muscle(s). Indeed, we (Brownstein et al., 2019, 2017) and others (Rampinini et al., 2011; Thomas et al., 2017) have found that reductions in maximal voluntary contraction (MVC) force of the knee extensors persist for 48–72 h following football match‐play. Reductions in MVC can be attributed to peripheral mechanisms, involving impairments in contractile function owing to disturbances in the excitation–contraction coupling process and/or myofibrillar force production (Allen, 2001; Allen et al., 2008), and a central mechanism, involving a reduction in nervous system voluntary activation (VA) of muscle (Gandevia, 2001). Impairments in contractile function can be determined by measuring involuntary evoked responses to electrical stimulation at rest, while VA can be examined through evoked responses to electrical stimulation during MVCs (Millet et al., 2011). Using these methods, we found that impairments in both contractile function and VA persisted for 48–72 h following match‐play (Brownstein et al., 2019, 2017; Thomas et al., 2017). Thus, both central and peripheral mechanisms contribute to the prolonged impairments in neuromuscular function following team sport activity, with these impairments likely occurring as a manifestation of the aforementioned metabolic and histological perturbations associated with team sport activity.

At present, there are limited data on whether impairments in neuromuscular function are accentuated when bouts of high‐intensity intermittent team sport exercise are performed with incomplete recovery. Although several studies have found that reductions in neuromuscular function are exacerbated when brief bouts of metabolically demanding exercise are interspersed with brief recovery periods, such as in response to repeated sprints (Di Domenico et al., 2022; Goodall et al., 2015), high‐intensity intervals (Decorte et al., 2012) or following neuromuscular electrical stimulation (Hureau et al., 2014), no study has assessed neuromuscular function when prolonged metabolically and mechanically strenuous exercise is repeated across days with incomplete recovery. Such a study is warranted given the importance of task‐dependency in determining the aetiology and magnitude of impaired neuromuscular function (Enoka, 1995), and that different mechanisms contribute to impaired neuromuscular function during prolonged metabolically and mechanically demanding exercise compared with brief metabolically demanding exercise (Brownstein et al., 2021). Moreover, given that congested fixture schedules are commonplace in team sports such as football (Julian et al., 2021), such a study might also be of relevance to practitioners working with team sport athletes.

The aim of the present study was to assess the effect of performing two bouts of prolonged metabolically and mechanically strenuous exercise separated by 48 h on neuromuscular function and the perceptual, heart rate and metabolic responses in competitive football players. It was hypothesised that impairments in neuromuscular function would be exacerbated following the second bout relative to the first due to commencing exercise with persistent impairments in neuromuscular function.

## 2 METHODS

### 2.1 Ethical approval

All participants provided written informed consent prior to commencing testing. The study received ethical approval from the Newcastle University Faculty of Medical Sciences Research Ethics committee (ethical approval number: 2420/25146) in accordance with the ethical standards established in the *Declaration of Helsinki*, apart from registration in a database.

### 2.2 Participants

A convenience sample of 12 amateur competitive football players (age 22 ± 3 years, stature 181.4 ± 5.1 cm, body mass 70.9 ± 6.1 kg), including one female, provided written informed consent to take part in the study. Self‐reported match‐play and training hours per week were 1.8 ± 0.9 and 3.4 ± 1.6 h, respectively. The study was conducted from October to March, during the competitive season when players were accustomed to their normal training and game load. Participants were asked to refrain from strenuous exercise in the 48 h prior to the first experimental visit, and between the first and second experimental visit. Moreover, participants were asked to arrive at the laboratory 2–3 h post‐prandial, and recorded their nutritional intake on the day of the first experimental visit and subsequently replicated the content and timing of food intake on the day of the second experimental visit.

### 2.3 Design

**FIGURE 1:**
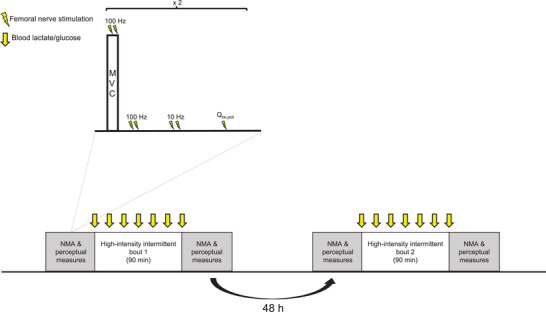
Schematic representation of the study protocol and neuromuscular assessment. MVC, maximal voluntary contraction; NMA, neuromuscular assessment; *Q*_tw,pot_, potentiated twitch torque.

A schematic representation of the study design is shown in Figure 1. During the initial visit, participants attended the laboratory to perform a multi‐stage fitness test and familiarisation with the experimental procedures prior to the experimental visits. During the subsequent two visits, participants performed two ∼90 min bouts of a modified team sport match simulation separated by 48 h. Bouts of exercise were performed on an indoor synthetic running track, in a temperature‐controlled environment. Prior to and following both exercise bouts, measures of neuromuscular function, fatigue and soreness were taken. During both matches, rate of perceived exertion (RPE), blood lactate, blood glucose, sprint time and heart rate (HR) were recorded.

### 2.4 Procedures

#### 2.4.1 Multi‐stage fitness test and familiarisation

During the initial visit to the laboratory, participants completed a multi‐stage fitness test to measure aerobic fitness and to determine appropriate running velocities for the modified team sport match protocol (Nicholas et al., 2000). Subsequently, participants were familiarised with the neuromuscular and perceptual measured employed in the study, before completing 5 min of the high‐intensity intermittent exercise protocol.

#### 2.4.2 High‐intensity intermittent exercise protocol

On the days of the first simulated match (bout 1), participants completed ∼90 min of the modified Loughborough Intermittent Shuttle Test (LIST) (Nicholas et al., 2000). Prior to the LIST, participants completed measures of neuromuscular function, fatigue, soreness, blood glucose and blood lactate (all described below), before performing a 5 min standardised warm‐up. The warm‐up consisted of jogging at varying speeds of 10–12 km/h as well as dynamic mobility exercises. The LIST protocol consists of 2 × ∼45 min halves of exercise at varying intensities separated by 15 min recovery, with each half consisting of 3 × ∼15 min blocks separated by 3 min recovery. The exercise within each block consisted of 10 repetitions of walking (3 × 20 m), jogging (3 × 20 m) at 55% of maximum speed from the multi‐stage fitness test, and running (3 × 20 m) at 95% of maximum speed from the multi‐stage fitness test, with speeds guided by an audible beep, as well as one maximum 20 m sprint. This protocol has been shown to induce similar physiological responses to football match‐play (Nicholas et al., 2000). The protocol was modified to include forced decelerations to a target line situated 3 m from the finish following each 20 m sprint, similar to Thomas et al. (2017). This procedure was used to elicit muscle damage and ensure that impairments in neuromuscular function persisted for at least 48 h following bout 1. Heart rate and 20 m sprint time were recorded throughout the exercise bouts, while measures of blood lactate, blood glucose and RPE were recorded following each block (described below). Two min following the end of the bout, the neuromuscular assessment began and blood lactate and blood glucose measures were taken, followed by measures of fatigue and soreness, in the described order. This entire protocol was then precisely replicated 48 h later for bout 2.

#### 2.4.3 Perceptual measures

Fatigue and soreness were measured using visual analogue scales, with the latter used as an indicator of muscle damage. Participants were required to draw a vertical line on a 100 mm horizontal line in response to the questions, ‘How fatigued do you feel’ and ‘How sore do your muscles feel’. The visual analogue scales were anchored on either side with verbal descriptors ‘not at all’ to ‘extremely’. Rate of perceived effort was measured at the end of each 15 min block, with participants asked ‘how much effort did you exert during the previous block?’ before responding using the Borg 6‐20 RPE scale (Borg, 1982).

#### 2.4.4 Assessment of neuromuscular function

Neuromuscular assessments were performed on the right knee extensor and consisted of a maximal voluntary contraction (MVC) with a superimposed high‐frequency doublet (100 Hz), potentiated high‐ and low‐frequency (10 Hz) doublets delivered by electrical stimulation to the femoral nerve while relaxed (Db100 and Db10, respectively), and a single potentiated twitch delivered by electrical stimulation to the femoral nerve while relaxed (*Q*_tw,pot_; Figure 1). The knee extensors were studied as this muscle group incurs significant decrements in function as a result of team sport activity (Brownstein et al., 2017; Thomas et al., 2017), and the moderate to excellent reliability of variables from the neuromuscular assessment employed in the present study has been repeatedly demonstrated (Brownstein et al., 2022; Thomas et al., 2016). The pre‐bout 1 and pre‐bout 2 assessments began with the determination of the appropriate intensity for electrical nerve stimulation (described below). Subsequently, a warm‐up consisting of contraction at 25%, 50%, 75% and 90% of perceived maximum force was performed. Afterward, participants performed 2 × 3 s MVCs without stimulation. Next, 2 × 3 s MVCs with superimposed doublets were performed, followed by Db100, Db10 and *Q*_tw,pot_ at 2, 4 and 6 s post‐contraction, respectively. One minute separated all baseline MVCs. For the post‐bout 1 and post‐bout 2 neuromuscular assessments, only two MVCs with superimposed and post‐contraction stimuli were performed, separated by 10 s. The variables derived from the neuromuscular assessment included MVC, high‐frequency torque (Db100), low‐to‐high‐frequency torque (Db10:100), potentiated twitch torque (*Q*_tw,pot_), the maximum compound muscle action potential (M_max_) measured using electromyography (EMG, described below) and voluntary activation (VA).

#### 2.4.5 Heart rate and capillary blood sample

Heart rate was measured continuously throughout bout 1 and bout 2 using a Polar HR monitor attached to a chest strap (Polar H10, Polar Electro Oy, Helsinki, Finland). Mean and peak HR were determined for each 15 min block. Capillary blood samples were taken at the end of each 15 min block to measure blood lactate and glucose concentration (Biosen C‐Line Clinic, EKF Diagnostics, Cardiff, UK).

#### 2.4.6 Sprint duration

During each sprint throughout the high‐intensity intermittent protocols, 20 m sprint time was recorded using electronic timing gates (Witty Gate, Microgate, Bolzano, Italy). Sprints were initiated from a standing start 30 cm behind the first timing gate, with participants encouraged to sprint maximally through the timing gate at 20 m, before decelerating as rapidly as possible. Total sprint time for each block was calculated as the sum of the 10 sprints in each block, with minimum sprint time for each block also analysed.

### 2.5 Instrumentation

#### 2.5.1 Torque and electromyography

A dynamometer was used to measure knee extensor torque (N m) during voluntary and evoked contractions (Biodex System 4 Pro, Biodex Medical Systems Inc., Shirley, NY, USA). Torque was transmitted through a non‐compliant cuff strongly fixed above the malleoli. The position of the chair for each participant was recorded during the initial visit and was kept consistent for subsequent visits. Participants were attached to the chair using two seatbelts across the chest and were asked to cross their arms during contractions to minimise movement of the torso. During contractions, knee angle was 90° flexion and hip angle 60° flexion. Electrical activity from the vastus lateralis was recorded from surface EMG electrodes (10 mm recording diameter; Meditrace 100, Covidien, Dublin, Ireland) with a 30 mm inter‐electrode distance. A reference electrode was placed on the patella. Electrode placement was marked with indelible ink to ensure consistent placement of electrodes during neuromuscular measurements. Before applying the electrodes, the skin was shaved, gently abraded and cleaned using isopropyl alcohol. The EMG signals were amplified with an octal bioamplifier (FE231, ADInstruments, Bella Vista, Australia), bandpass filtered (10–500 Hz) and analog‐to‐digitally converted at a sampling rate of 2000 Hz using a PowerLab system (PowerLab 8/35, ADInstruments).

#### 2.5.2 Femoral nerve stimulation

Stimulations of the right femoral nerve were delivered via a constant‐current stimulator (DS7R, Digitimer, Welwyn Garden City, UK) by means of a 30 mm‐diameter surface cathode (Meditracte 100) placed on the femoral triangle and a 50 × 100 mm anode (Easysnap, Compex, Guildford, UK) placed above the gluteal fold. Single rectangular electrical pulses of 1 ms duration and 400 V maximal output voltage were delivered. During the pre‐bout 1 and 2 assessment, electrical stimuli were first administered at 30 mA and were then increased in 30 mA increments until the maximum quadriceps twitch amplitude and M_max_ were elicited. The resulting stimulation intensity was then increased by 30% to account for activity‐induced changes in axonal excitability, with this same intensity used for post‐bout 1 and 2 neuromuscular assessments.

### 2.6 Data analysis

The peak‐to‐peak amplitude of the MVC and M_max_ were recorded and used for analysis. The ratio of the evoked torque response to Db10 and Db100 (Db10:100) was used to determine low‐frequency force depression. For the assessment of VA, the following equation was used (Merton, 1954):

$$\%\mathrm{VA}\;=\;\left(1\;-\frac{\mathrm{SIT}}{\mathrm{Db}100}\;\right)\times 100$$

When stimuli were not delivered at the peak torque, a correction was applied using the torque at stimulation (*T*_atstim_), the peak torque, the size of the superimposed twitch (SIT), and the Db100, with the following equation applied (Strojnik & Komi, 1998):

$$\%{\mathrm{VA}}_{\mathrm{corrected}}=\;1-\left(\mathrm{SIT}\times \left(\frac{T_{\mathrm{atstim}}}{\mathrm{MVC}}\right)/\mathrm{Db}100\right)\times 100$$

### 2.7 Statistical analysis

Jamovi statistical software (Jamovi, version 1.0, 2019, the jamovi project; retrieved from https://www.jamovi.org) was used for all statistical analyses. All data are presented as means ± SD. Statistical significance was set at an α‐level of 0.05. Normality of the data was assessed by the Shapiro–Wilk test, with no data requiring transformation. Assumptions of sphericity were explored and controlled for all variables with the Greenhouse–Geisser adjustment, where necessary. For RPE, blood lactate, blood glucose, total and minimum sprint speed, a two‐way repeated measures ANOVA was used (bout × time). For the neuromuscular, fatigue and soreness measures, a one‐way repeated measures ANOVA was used to assess changes across time (pre‐bout 1, post‐bout 1, pre‐bout 2 and post‐bout 2). In the event of a significant main effect or interaction, a Tukey post‐hoc analysis was performed to locate where differences lay. Partial eta squared (η_p_^2^; ANOVA) was calculated to estimate effect sizes, with values representing small (<0.13), medium (≥0.13, <0.26) and large (≥0.26) (Cohen, 1988). Standardised effect sizes (Cohen's *d*) were calculated for focused pairwise comparisons and interpreted as small (≥0.2), moderate (≥0.5) and large (≥0.8) (Cohen, 1988).

## 3 RESULTS

### 3.1 Neuromuscular function

**FIGURE 2:**
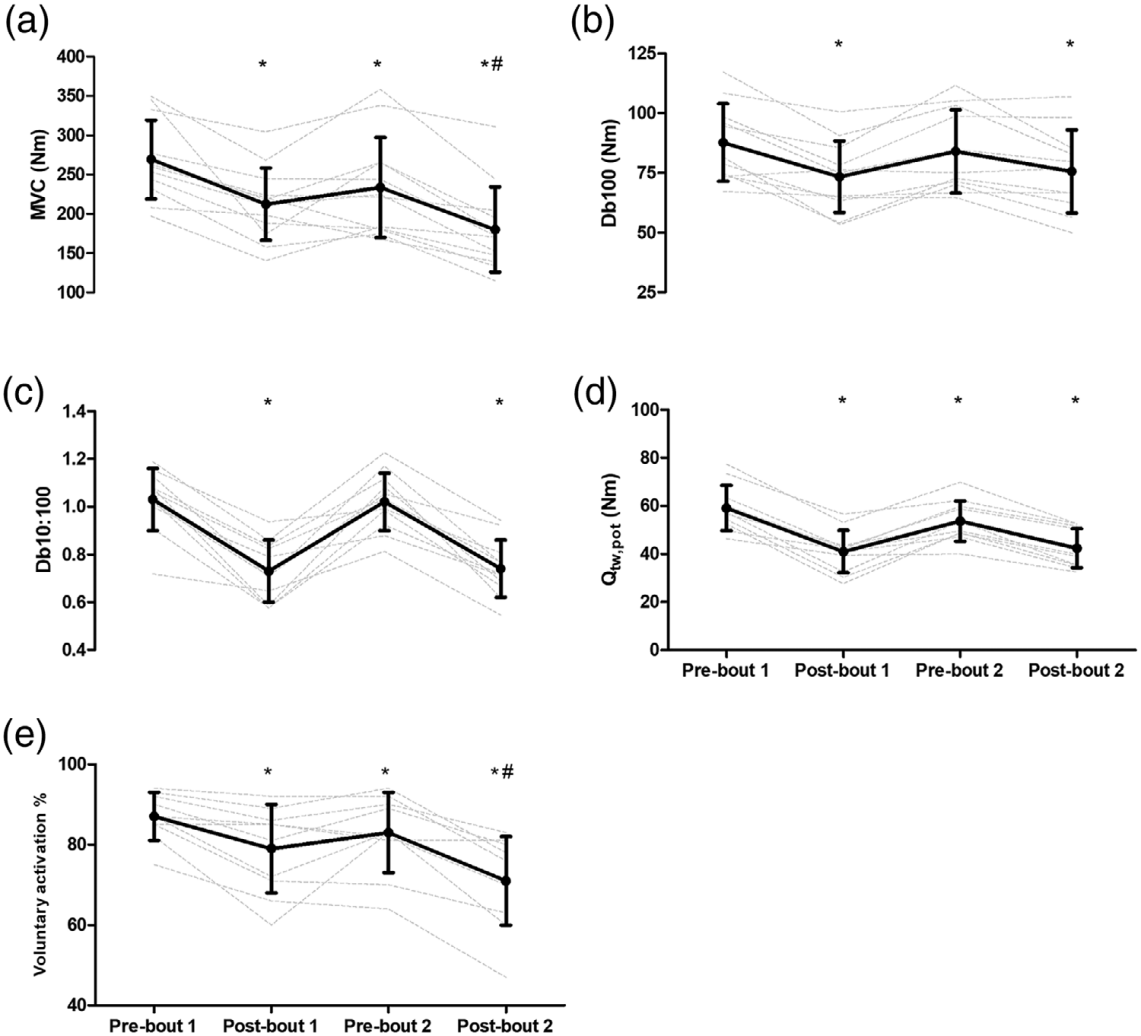
Maximal voluntary contraction (MVC, a), high‐frequency doublet (Db100, b), low‐ to high‐frequency torque ratio (Db10:100, c), potentiated twitch torque (*Q*_tw,pot_, d) and voluntary activation (e) measured pre‐ and post‐bouts of high‐intensity intermittent exercise separated by 48 h (*n* = 12). *Significant differences in comparison with pre‐bout 1, *P* < 0.05; #significant differences in comparison with post‐bout 1, *P* < 0.05. Continuous lines are means ± SD; dashed lines show individual responses.

Relative to pre‐bout 1, MVC was reduced by 21 ± 12% post‐bout 1 (*P* = 0.003, *d* = 1.18), 14 ± 11% pre‐bout 2 (*P* = 0.009, *d* = 0.62) and 33 ± 14% post‐bout 2 (*P* < 0.001, *d* = 1.71; time effect: *F*_3,33_ = 24.3, *P* < 0.001, η_p_^2^ = 0.69). Maximal voluntary contraction was 15 ± 18% lower post‐bout 2 than post‐bout 1 (*P* = 0.036, *d* = 0.64; Figure 2a). There was a 16 ± 11% reduction in Db100 from pre‐ to post‐bout 1 (*P* = 0.004, *d* = 0.92), which recovered by pre‐bout 2 (*P* = 0.572, *d* = 0.22) and was again reduced by 14 ± 13% relative to pre‐bout 1 at post‐bout 2 (*P* = 0.035, *d* = 0.72; time effect: *F*_3,33_ = 10.5, *P* < 0.001, η_p_^2^ = 0.51). There was no difference between Db100 at post‐bout 1 and post‐bout 2 (*P* = 0.850, *d* = 0.14; Figure 2b). The Db10:100 was reduced by 29 ± 11% from pre‐ to post‐bout 1 (*P* < 0.001, *d* = 2.31), recovered by pre‐bout 2 (*P* = 0.970, *d* = 0.08) and was reduced by 28 ± 9% post‐bout 2 relative to pre‐bout 1 (*P* < 0.001, *d* = 2.32; time effect: *F*_3,33_ = 43.9, *P* < 0.001, η_p_^2^ = 0.82). There was no difference between Db10:100 at post‐bout 1 and post‐bout 2 (*P* = 0.993, *d* = 0.08; Figure 2c). For *Q*_tw,pot_, there was a reduction of 30 ± 11% from pre‐ to post‐bout 1 (*P* < 0.001, *d* = 1.98), which remained below baseline by 9 ± 6% at pre‐bout 2 (*P* = 0.004, *d* = 0.60) and was 28 ± 8% lower than baseline at post‐bout 2 (*P* < 0.001, *d* = 1.91; time effect: *F*_3,33_ = 53.0, *P* < 0.001, η_p_^2^ = 0.84). There was no difference in *Q*_tw,pot_ at post‐bout 1 and post‐bout 2 (*P* = 0.872, *d* = 0.17; Figure 2d). The torque level at stimulation during VA measurements did not differ across time (*F*_3,33_ = 2.3, *P* = 0.096, η_p_^2^ = 0.17), and was 89 ± 7%, 89 ± 9%, 83 ± 10% and 85 ± 9% for pre‐ and post‐bout 1 and pre‐ and post‐bout 2, respectively. When stimulations were not delivered at peak torque, a correction equation was applied (see Methods). For VA, there was an 8 ± 7% reduction from pre‐ to post‐bout 1 (*P* = 0.023, *d* = 0.90), with VA recovering by pre‐bout 2 (*P* = 0.133, *d* = 0.49), and again being reduced relative to baseline by 16 ± 7% at post‐bout 2 (*P* < 0.001, *d* = 1.81; time effect: *F*_3,33_ = 20.2, *P* < 0.001, η_p_^2^ = 0.69). Voluntary activation was lower post‐bout 2 than post‐bout 1 by 8 ± 7% (*P* = 0.029, *d* = 0.73; Figure 2e). There was no change in M_max_ amplitude across time (*F*_3,33_ = 0.2, *P* = 0.926, η_p_^2^ = 0.01).

### 3.2 Heart rate and metabolic responses

**FIGURE 3:**
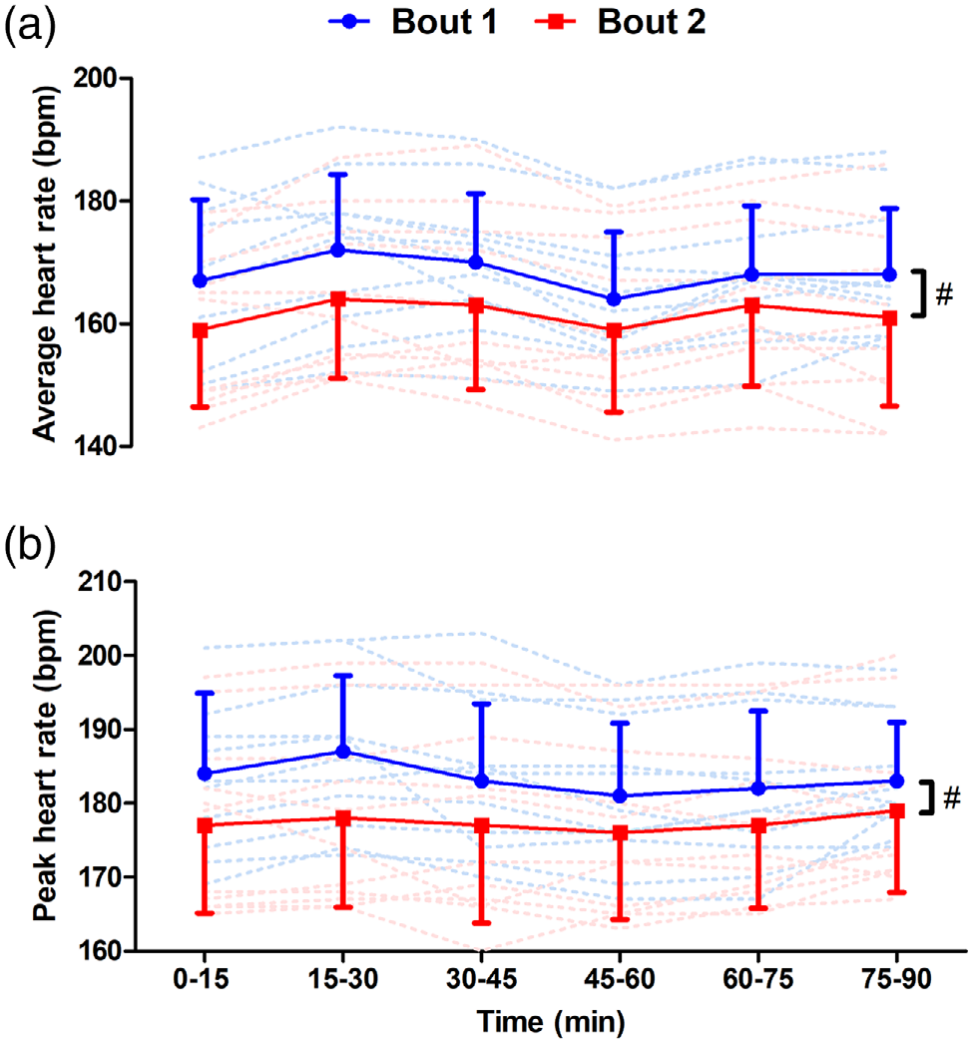
Mean (a) and peak (b) HR measured during bouts of high‐intensity intermittent exercise separated by 48 h (*n* = 12). #Main effect of bout, *P* < 0.05; *difference in peak HR between bouts 1 and 2 at corresponding time‐point, *P* < 0.05. Continuous lines are means ± SD; dashed lines show individual responses for bout 1 (blue) and bout 2 (red).

Mean and peak HR during each block throughout bouts 1 and 2 are displayed in Figure 3. Mean HR was lower throughout bout 2 than bout 1, with a main effect of bout (*F*_1,11_ = 7.5, *P* = 0.021, η_p_^2^ = 0.43), and no bout × time interaction (*F*_5,55_ = 1.0, *P* = 0.403, η_p_^2^ = 0.09). Peak HR was also lower throughout bout 2 than bout 1, with a main effect of bout (*F*_1,11_ = 22.5, *P* < 0.001, η_p_^2^ = 0.69). A bout × time interaction was also found (*F*_5,55_ = 3.4, *P* = 0.010, η_p_^2^ = 0.26), with peak HR lower at block 1 and block 2 for bout 2 compared to the same time points for bout 1 (*P* ≤ 0.008).

**FIGURE 4:**
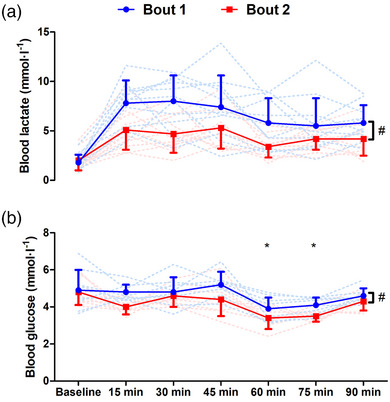
Blood lactate (a) and blood glucose (b) measured during bouts of high‐intensity intermittent exercise separated by 48 h (*n* = 12). #Main effect of bout, *P* < 0.05; *time effect for differences compared to baseline, *P* < 0.05. Continuous lines are means ± SD; dashed lines show individual responses for bout 1 (blue) and bout 2 (red).

Blood lactate was lower throughout bout 2 than bout 1 (*F*_1,11_ = 25.1, *P* < 0.001, η_p_^2^ = 0.72). A bout × time interaction for blood lactate was also found (*F*_6,66_ = 3.2, *P* = 0.009, η_p_^2^ = 0.24). For bout 1, blood lactate was higher than pre‐bout 1 following all blocks apart from block 5 (all *P* ≤ 0.014). For bout 2, blood lactate was higher than pre‐bout 2 following blocks 1, 2 and 5 (all *P* ≤ 0.030), with no difference at blocks 3, 4 or 6 (Figure 4a). For blood glucose, there was a main effect of time (*F*_2.9,20.2_ = 10.1, *P* < 0.001, η_p_^2^ = 0.59), with blood glucose lower than baseline following blocks 4 and 5 (both *P* ≤ 0.041). Blood glucose was lower throughout bout 2 than bout 1 (*F*_1,11_ = 11.4, *P* = 0.012, η_p_^2^ = 0.62). There was no bout × time interaction (*F*_6,66_ = 1.2, *P* = 0.327, η_p_^2^ = 0.15; Figure 4b).

### 3.3 Sprint duration

**FIGURE 5:**
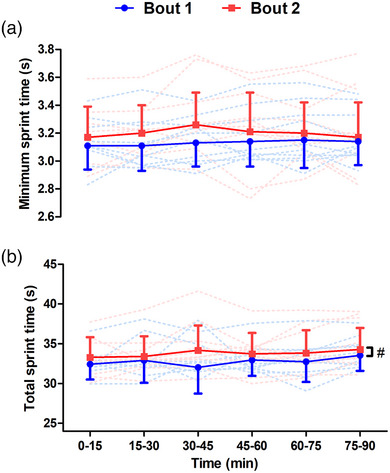
Minimum (a) and total (b) sprint time measured during bouts of high‐intensity intermittent exercise separated by 48 h (*n* = 12). #Main effect of bout, *P* < 0.05. Continuous lines are means ± SD; dashed lines show individual responses for bout 1 (blue) and bout 2 (red).

There was no difference in minimum sprint time between bouts 1 and 2, with no effect of bout (*F*_1,11_ = 4.2, *P* = 0.070, η_p_^2^ = 0.32) and no bout × time interaction (*F*_6,66_ = 0.9, *P* = 0.468, η_p_^2^ = 0.09; Figure 5a). Total sprint time was higher throughout bout 2 than bout 1 (*F*_1,11_ = 11.5, *P* = 0.008, η_p_^2^ = 0.56), with no bout × interaction (*F*_2.2,19.4_ = 0.4, *P* = 0.704, η_p_^2^ = 0.04; Figure 5b).

### 3.4 Perceptual responses

**FIGURE 6:**
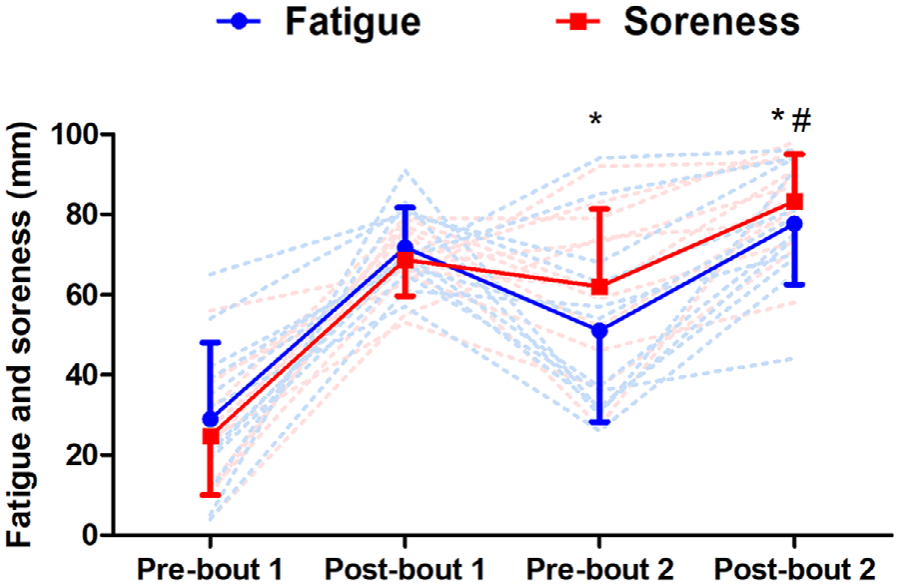
Fatigue and soreness measured pre‐ and post‐bouts of high‐intensity intermittent exercise separated by 48 h (*n* = 12). *Difference from pre‐bout 1 for both fatigue and soreness, *P* < 0.05; #difference from post‐bout 1 for soreness, *P* < 0.05. Continuous lines are means ± SD; dashed lines show individual responses for fatigue (blue) and soreness (red).

**FIGURE 7:**
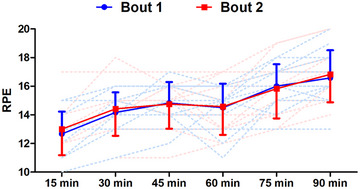
Rate of perceived exertion measured during bouts of high‐intensity intermittent exercise separated by 48 h (*n* = 12). Continuous lines are means ± SD; dashed lines show individual responses for bout 1 (blue) and bout 2 (red).

Fatigue was elevated from pre‐ to post‐bout 1 (*P* < 0.001), and remained higher than baseline at both pre‐bout 2 (*P* = 0.013) and post‐bout 2 (*P* < 0.001; time effect: *F*_3,33_ = 30.2, *P* < 0.001, η_p_^2^ = 0.73). There was no difference in fatigue at post‐bout 1 and post‐bout 2 (*P* = 0.507; Figure 6). Soreness increased pre‐ to post‐bout 1 (*P* < 0.001), and remained higher than baseline at both pre‐bout 2 (*P* < 0.001) and post‐bout 2 (*P* < 0.001; time effect: *F*_3,33_ = 47.7, *P* < 0.001, η_p_^2^ = 0.81). Soreness was higher post‐bout 2 than post‐bout 1 (*P* = 0.005; Figure 6). A time effect was found for RPE (*F*_3,33_ = 19.4, *P* < 0.001, η_p_^2^ = 0.64), which was higher than block 1 at blocks 2–6 (all *P* ≤ 0.020). There was no difference in RPE between bouts 1 and 2, with no main effect of bout (*F*_1.8,19.7_ = 0.2, *P* = 0.693, η_p_^2^ = 0.15) and no bout × time interaction (*F*_6,66_ = 0.4, *P* = 0.862, η_p_^2^ = 0.03; Figure 7).

## 4 DISCUSSION

The aim of the present study was to assess neuromuscular function and the perceptual, heart rate and metabolic responses to two bouts of prolonged metabolically and mechanically strenuous exercise separated by 48 h. The first bout (bout 1) elicited large impairments in neuromuscular function which manifested in reduced knee extensor maximal force (MVC). The reduced MVC persisted 48 h later at pre‐bout 2, and the reductions were then greater post‐bout 2 than post‐bout 1. Although contractile function, measured through resting twitch responses, was impaired following both bouts, there was no difference between the reduction in contractile function post‐bout 2 relative to post‐bout 1. In contrast, the reduction in voluntary activation (VA) was greater following bout 2 than bout 1. Thus, reductions in knee extensor neuromuscular function are exacerbated when bouts of high‐intensity intermittent exercise with high mechanical and metabolic demands are performed with incomplete recovery, with the accentuated reductions in MVC likely due to impairments in nervous system VA of the knee extensors. The heart rate and metabolic responses to high‐intensity intermittent exercise performed with incomplete recovery were also altered. Specifically, mean and peak HR were lower during bout 2 compared with bout 1, likely due to the lower sprint speed during bout 2 meaning that the overall intensity of the latter bout was reduced. Finally, blood lactate and blood glucose concentrations were lower during bout 2 than bout 1, which might be due, at least in part, to reduced glycogen availability upon commencing exercise and consequently a greater reliance on glucose extraction. These results provide novel insight into the effects of performing metabolically and mechanically demanding high‐intensity intermittent exercise with incomplete recovery on neuromuscular function and the heart rate and metabolic responses to exercise.

The present study utilised a modified version of the LIST protocol in order to induce substantial and sustained impairments in neuromuscular function. Following bout 1, MVC was reduced by 21 ± 12%, concurrent with a 30 ± 11% reduction in *Q*_tw,pot_ and an 8 ± 11% reduction in VA. While the reductions in MVC and VA were comparable to the 17% and 8% reductions, respectively, found by Thomas et al. (2017) using a simulated match protocol, our reduction in *Q*_tw,pot_ was substantially greater than their 14% reduction. This can likely be explained, at least in part, by the greater number of sprints with forced decelerations used in the present study than in Thomas et al. (2017) (60 and 42 sprints, respectively) and/or differences in the playing level of the participants (amateur and semi‐professional football players, respectively). Following 48 h of recovery, at pre‐bout 2, MVC remained below baseline by 14 ± 12%, which was considerably greater than the 7% reduction at the same time point found by Thomas et al. (2017), and occurred concurrently with persistent reductions in *Q*_tw,pot_. Thus, the initial bout of high‐intensity intermittent exercise elicited substantial and sustained reductions in neuromuscular function, such that participants began bout 2 having recovered incompletely.

A key and novel finding from the present study was that having begun bout 2 with persistent impairments in neuromuscular function, these impairments were exacerbated post‐bout 2 relative to post‐bout 1. Specifically, MVC was 15 ± 18% lower following bout 2 than following bout 1, representing a moderate effect size. The results indicate that the accentuated reduction in MVC was a result of exacerbated reductions in VA. While impairments in contractile function were present following bout 2, as demonstrated by the 28 ± 8% reduction in *Q*_tw,pot_ and the decreases in Db100 and Db10:100 relative to baseline, these reductions were all not different from those found following bout 1. In contrast, the reduction in VA was 8 ± 7% greater post‐bout 2 than post‐bout 1, also representing a moderate effect size. These are the first data, to our knowledge, showing that impairments in neuromuscular function can accumulate when metabolically and mechanically strenuous exercise is repeated with incomplete recovery following exercise in the preceding days, and suggest that deficits in nervous system activation of muscle are likely responsible for the exacerbated reduction in neuromuscular function.

The impairments in contractile function observed following both bouts can likely be attributed to metabolite accumulation (Allen et al., 2008; Debold et al., 2016), muscle damage (Allen, 2001) and/or glycogen depletion (Ørtenblad et al., 2013), all of which occur to a large extent during high‐intensity intermittent exercise (Krustrup et al., 2022, 2011; Mohr et al., 2016) and disturb the excitation–contraction coupling and/or myofibrillar force production. The finding that reductions in resting twitch responses *Q*_tw,pot_, Db100 and Db10:100 were similar following both bouts suggests that these disturbances were not exacerbated by the second bout, despite the attenuated contractile function upon commencing bout 2. This finding might be explained, at least in part, by the lower intensity of the second bout. Specifically, total sprint time was higher during bout 2 (i.e., sprint speed was slower), which likely contributed to the lower mean and peak HR and, at least in part, blood lactate during the same bout. This slower sprint speed could be explained by the reduced maximal neuromuscular performance upon commencing the second bout and/or the adoption of a pacing strategy during the bout 2 sprints (Billaut et al., 2011), particularly as fatigue and soreness were elevated at the beginning of bout 2. In turn, the lower intensity of the second bout would be expected to lead to a lower accumulation of contractile function‐impairing metabolites and reduce the force of the eccentric contractions associated with the forced decelerations following sprinting. These results are similar to those of numerous studies showing that reductions in *Q*_tw,pot_ reach a nadir during a single bout of a team sport match simulation (Goodall, Thomas, Harper, et al., 2017), high‐intensity intermittent cycling (Decorte et al., 2012) and repeated sprints (Goodall et al., 2015), including those performed in a ‘pre‐fatigued’ state following neuromuscular electrical stimulation (Hureau et al., 2014). Accordingly, in line with previous findings, reductions in contractile function are not accentuated during prolonged high‐intensity intermittent exercise when commencing exercise with contractile function in an impaired state.

It has long been established that during a single bout of exercise, reductions in VA increase with the amount of work completed (Brownstein et al., 2021; Millet et al., 2018; Thomas et al., 2016, 2015). The finding of exacerbated losses in VA following bout 2 in the present study confirms that the same is also true for exercise performed on separate days with incomplete recovery following a previous bout of exercise. The mechanisms contributing to reduced VA during prolonged exercise are, however, unknown. Studies measuring VA using transcranial magnetic stimulation suggest that part of the reduction in VA might be due to suboptimal output from the motor cortex (Søgaard et al., 2006), but the contributing mechanisms are uncertain. A role for group III/IV afferents, which inhibit motor cortical cells and reduce VA (Sidhu et al., 2018), can be speculated given that the discharge of these afferents increases in response to elevated biochemical and histological substances associated with metabolic stress, muscle damage and inflammation (Kaufman et al., 1983; Mense, 1977), which together could act synergistically to elevate afferent discharge (Light et al., 2008). Given that group III/IV afferents are thought to be the sole source of pain from skeletal muscle in response to nociceptive stimuli (Graven‐Nielsen et al., 2004; McCord & Kaufman, 2010), this suggestion is supported by perceptions of muscle soreness being higher following bout 2 than bout 1. Notwithstanding the mechanistic uncertainty, these results indicate that impairments in nervous system function are responsible for accentuated reductions in neuromuscular function when metabolically and mechanically strenous high‐intensity intermittent exercise is performed with incomplete recovery.

A further finding of interest in the present study was that, despite the exacerbated impairments in neuromuscular function following bout 2, perceptions of fatigue were not increased post‐bout 2 relative to post‐bout 1. Moreover, RPE was not different between bouts, despite commencing bout 2 with elevated fatigue, soreness and impairments in neuromuscular function. This finding might again be explained by the lower intensity of bout 2 owing to the slower sprint speed. One possibility is that participants paced their efforts to a greater extent during sprints throughout bout 2 in order to ensure perceptions of fatigue and effort were maintained within tolerable limits. Indeed, it has previously been demonstrated that a degree of pacing occurs during repeated sprints (Billaut et al., 2011), and, given the high volume of sprints in the present study and the elevated fatigue and soreness upon commencing bout 2, it seems plausible that pacing strategies could have been adopted to prevent intolerable levels of fatigue and effort during the second bout.

The present study assessed blood lactate and blood glucose concentrations at 15 min intervals throughout both bouts of high‐intensity intermittent exercise. The time effect demonstrating lower blood glucose at 60 and 75 min of exercise is similar to previous findings (Bangsbo et al., 2007; Russell et al., 2011). This is thought to be the result of continued glucose uptake by skeletal muscle during the 15 min half‐time rest interval without the feedforward stimulation of hepatic glucose release (Bangsbo et al., 2007; Wasserman & Cherrington, 2011). A further interesting and novel finding from the present study was that both blood lactate and blood glucose were lower during bout 2 than bout 1. For example, during bout 2, blood glucose dropped to concentrations as low as 3.4 ± 0.6 mmol/l, whereas minimum blood glucose was 3.9 ± 0.6 mmol/l during bout 1. The lower blood lactate concentration during bout 2 can likely be explained, at least in part, by the lower intensity relative to bout 1, as indicated by the higher total sprint time and lower mean and peak HR. However, the concurrently lower blood lactate and glucose concentrations point towards lower muscle glycogen stores upon commencing bout 2. Indeed, previous research has demonstrated that muscle glycogen stores remain lower than pre‐exercise levels 48 h following competitive football match‐play (Gunnarsson et al., 2013; Krustrup et al., 2011). Given that the prolonged high‐intensity intermittent nature of the modified LIST protocol in the present study, and that substantial muscle damage, which impairs glycogen resynthesis (Asp et al., 1995), was likely incurred via the high volume of sprints with forced decelerations, it is expected that glycogen stores remained depressed going into bout 2. In turn, it is well established that reliance on blood glucose extraction increases when performing exercise in a glycogen depleted state (Steensberg et al., 2002; Wojtaszewski et al., 2003), possibly explaining the lower blood glucose throughout bout 2 in our study. Despite a possibly greater glucose extraction during the second bout, the lower blood lactate concentrations suggest a lower glycolytic contribution to energy demands during bout 2. From a practical perspective, these results highlight the importance of nutritional interventions targeting restoration of glycogen stores in the intervening period between bouts of high‐intensity intermittent exercise performed with limited recovery, as well as the preservation of blood glucose through carbohydrate ingestion during exercise bouts performed following incomplete recovery.

### 4.1 Limitations

One limitation of the present study was the 2 min delay between exercise cessation and the neuromuscular assessment. Consequently, the impairments in neuromuscular function following the exercise bouts were likely underestimated to some degree. Moreover, although the study was advertised to both males and females, the respondent rate of females was low, with only one female included in the present study, precluding a sex comparison. Given that previous studies have demonstrated lower fatigability in females compared with males in response to whole‐body locomotor exercise (Ansdell et al., 2020; Besson et al., 2021), future studies should consider the effect of sex on fatigability following damaging high‐intensity intermittent exercise. Finally, the study employed a correction equation to calculate VA when stimulations were not delivered at maximal torque during an MVC (Strojnik & Komi, 1998). While this equation assumes a linear relationship between the SIT and relative torque level, previous research measuring responses in the knee extensors has shown that this relationship deviates from linearity when stimulations are delivered at lower (<∼75% MVC) versus higher (>∼75% MVC) torque levels, with a disproportionately smaller SIT being observed at higher torque levels (Kooistra et al., 2007). While there is likely some degree of inaccuracy associated with the correction equation, all stimulations in the present study were delivered at high torque levels ≥75% MVC, above which the SIT–torque relationship is approximately linear (Kooistra et al., 2007). Moreover, there were no differences in the torque at stimulation between time points. Accordingly, the reductions in VA appear genuine and in line with previous observations using similar exercise modalities (Brownstein et al., 2017; Thomas et al., 2017), and are unlikely to be due to methodological issues.

### 4.2 Conclusion

The present study demonstrates, for the first time, that impairments in neuromuscular function are accentuated when metabolically and mechanically strenuous high‐intensity intermittent exercise is repeated with incomplete recovery. The exacerbated reductions in nervous system voluntary activation of quadriceps muscles, coupled with the similar reductions in contractile function following the two bouts of exercise, indicate that impairments in nervous system function are responsible for the accentuated decreases in neuromuscular function. The results further show altered physiological responses to successive bouts of high‐intensity intermittent exercise interspersed with 48 h recovery, with heart rate lower during the second bout, likely due to the lower sprint speed. Thus, participants were unable to maintain the same intensity during the second bout, likely due to the persistent fatigue, soreness and impairments in neuromuscular function when commencing exercise. Finally, blood lactate and blood glucose concentrations were depressed during the second bout, which could be related to lower muscle glycogen stores inducing a higher reliance on blood glucose extraction, and an overall reduction in the glycolytic contribution to energy demands.

## AUTHOR CONTRIBUTIONS

Callum G. Brownstein conceived and designed the study; Joseph Hubbard and Jade Pitcairn performed experiments; Callum G. Brownstein, Joseph Hubbard and Jade Pitcairn analysed data and interpreted results of experiment; Joseph Hubbard and Jade Pitcairn drafted manuscript; Callum G. Brownstein edited and revised manuscript. All authors have read and approved the final version of this manuscript and agree to be accountable for all aspects of the work in ensuring that questions related to the accuracy or integrity of any part of the work are appropriately investigated and resolved. All persons designated as authors qualify for authorship, and all those who qualify for authorship are listed.

## CONFLICT OF INTEREST

No conflicts of interest, financial or otherwise, are declared by the authors.

## FUNDING INFORMATION

No funding was received for the present study.

## Data Availability

Data available upon request.
